# Baseline Clinical Predictors and Toxicological Drivers of In‐Hospital Poisoning Mortality in Resource‐Constrained Emergency Care: A Firth Penalized Regression Analysis

**DOI:** 10.1002/hsr2.73455

**Published:** 2026-10-27

**Authors:** Zewdu Hurisa, Ayalneh Demissie, Nebyu Mekonnen, Birhanu Asefa, Abera Girma, Helen Bekele, Tesfa Gebremeskel

**Affiliations:** ^1^ Department of Internal Medicine, College of Health Sciences Arsi University Asella Ethiopia; ^2^ Department of Public Health, College of Health Sciences Arsi University Asella Ethiopia; ^3^ ASTU Student Health Center Adama Science and Technology University Adama Ethiopia; ^4^ Department of Pediatrics and Child Health, College of Health Sciences Arsi University Asella Ethiopia

**Keywords:** acute pesticide poisoning, aluminum phosphide, emergency medicine, Ethiopia, firth logistic regression, Glasgow coma scale, in‐hospital mortality, retrospective cohort study, triage

## Abstract

**Background:**

Evidence regarding clinical characteristics and determinants of mortality among patients with acute poisoning in agricultural regions of Ethiopia remains limited. This study aimed to evaluate baseline clinical predictors associated with in‐hospital mortality among acutely poisoned patients in a low‐resource tertiary emergency department.

**Methods:**

A retrospective observational study was conducted among consecutive acute poisoning cases presenting to Asella Referral and Teaching Hospital (October 2022–September 2024). Of 372 screened cases, 7 patients who left against medical advice were excluded, yielding a final analytical cohort of 365 patients. Sociodemographic, toxicological, and clinical data were extracted from medical records. The primary outcome was in‐hospital mortality. Multivariable Firth penalized logistic regression was performed to minimize small‐sample bias given sparse outcome events (15 deaths). A sensitivity model additionally adjusted for sex and rural residence.

**Results:**

The median age of patients was 22.0 years (IQR: 19.0–27.0); 230 (61.8%) were aged 15–24 years, 199 (53.5%) were female, and 245 (65.9%) were rural residents. Insecticides were the predominant poisoning category (157; 42.2%). Aluminum Phosphide (AlP) exposure occurred in 43 (11.6%) patients, resulting in 12 deaths (case‐fatality rate: 27.9%; 95% CI 15.3%–43.7%). Overall in‐hospital mortality was 4.1% (15/365; 95% CI 2.3%–6.6%), with AlP accounting for 80.0% of all cohort deaths. In the primary multivariable Firth model, AlP exposure (aOR 30.48, 95% CI 7.88–117.95) and admission Glasgow Coma Scale < 15 (aOR 19.90, 95% CI 5.09–77.88) were strongly and independently associated with in‐hospital mortality. These associations remained robust in sensitivity analyses.

**Conclusions:**

Impaired consciousness at admission (GCS < 15) and Aluminum Phosphide exposure serve as critical baseline indicators for rapid, low‐cost risk stratification. While these exploratory parameters highlight high‐risk groups, prospective multi‐center validation across diverse hospital tiers is required before formal integration into clinical triage algorithms.

**Abbreviations**
AIC: Akaike Information Criterion
AlP: Aluminum Phosphide
aOR: Adjusted Odds Ratio
ARTH: Asella Referral and Teaching Hospital
AUC: Area Under the Curve
CI: Confidence Interval
GCS: Glasgow Coma Scale
IQR: Interquartile Range
LAMA: Left Against Medical Advice
LMIC: Low‐ and Middle‐Income Country
OR: Odds Ratio
PSS: Poisoning Severity Score
ROC: Receiver Operating Characteristic
SpO_2_
: Peripheral Oxygen Saturation
VIF: Variance Inflation Factor

## 1 Introduction

Acute poisoning represents a major global public health concern and a primary reason for emergency department admissions, contributing substantially to avoidable morbidity and mortality worldwide [1, 2, 3]. In low‐ and middle‐income countries (LMICs), particularly across Sub‐Saharan Africa and South Asia, the epidemiological profile of poisoning differs significantly from that of high‐income nations [4, 5]. While pharmaceutical overdoses and recreational substance toxicities predominate in high‐income settings, acute pesticide poisoning constitutes the overwhelming burden of severe toxicological emergencies and fatal outcomes in agricultural regions of LMICs [5, 6, 7]. Unregulated commercial access to highly hazardous agrochemicals, combined with limited toxicological treatment resources, makes rapid clinical risk stratification critical for triaging acute pesticide toxicity in emergency care [8, 9, 10].

In Ethiopia, an agrarian economy where a substantial proportion of the population engages in smallholder farming, acute poisoning is a critical public health and emergency care challenge [1, 2]. Evidence across Ethiopian regions demonstrates marked variation in the magnitude, toxicological patterns, and outcomes of poisoning [1, 2, 3, 11]. A comprehensive systematic review and meta‐analysis estimated the overall pooled in‐hospital mortality from acute poisoning in Ethiopia at 4.69%, identifying rural residence, delayed presentation, and specific high‐toxicity chemical exposures as primary drivers of adverse outcomes [1]. Subsequent regional investigations at tertiary and specialized facilities continue to highlight high case fatality rates alongside significant heterogeneity in toxic agent profiles [2, 3, 11, 12].

Among the various toxic agents encountered in agrarian clinical practice, metallophosphides—most notably aluminum phosphide (AlP)—represent an exceptionally lethal category [13, 14, 15]. Broadly utilized as an inexpensive and effective fumigant to protect stored grains against pest infestation, AlP releases toxic phosphine gas (PH_3_) upon contact with moisture or gastric acid [15, 16]. Phosphine gas inhibits mitochondrial cytochrome c oxidase, inducing severe cellular hypoxia, excessive reactive oxygen species generation, profound myocardial depression, acute vascular collapse, and rapid multiorgan failure [14, 17, 18]. Although high‐volume agrochemicals like organophosphates account for the majority of clinical presentations, AlP exhibits a disproportionately severe case‐fatality rate (27.9%), accounting for four‐fifths of all in‐hospital poisoning deaths in agricultural tertiary centers [13]. Because no specific antidote exists, clinical management relies entirely on rapid resuscitation and aggressive supportive care [16, 19].

Early clinical risk stratification is essential in resource‐constrained emergency settings to identify patients at immediate risk of deterioration [20, 21]. The Glasgow Coma Scale (GCS), originally developed for traumatic brain injury assessment, provides a simple, rapid, and objective bedside measure of neurological impairment [22]. Altered consciousness at emergency arrival often reflects severe systemic hypoxia, metabolic derangement, or direct central nervous system toxicity, and has been identified as a critical prognostic indicator across various acute toxicological presentations [1, 3, 21]. Conversely, the Poisoning Severity Score (PSS) offers a standardized framework for grading acute toxicity based on clinical features, its application in multivariable prognostic modeling requires careful methodological handling [23]. When the most severe score category (PSS 4) is defined by a fatal outcome, including PSS as a candidate predictor in mortality regression models introduces outcome incorporation (circular logic), artificially overestimating model predictive performance [23]. Therefore, evaluating baseline clinical parameters available strictly at initial presentation —such as GCS—provides a more methodologically sound approach to prognostic modeling [21].

While complex toxicological screening and multi‐parameter severity scoring systems are standard in high‐income settings, emergency departments across LMICs require rapid, resource‐sparing risk stratification tools [1, 2, 3]. Although existing systematic reviews provide valuable aggregated syntheses, empirical evidence on the use of penalization methods—particularly Firth's penalized logistic regression—to address sparse‐data artifacts and small event counts in low‐resource agricultural emergency departments remains limited [1, 2, 3]. Therefore, this study aimed to evaluate agent‐specific lethality and baseline clinical predictors of in‐hospital mortality among acute poisoning patients at a tertiary referral hospital in agricultural Ethiopia [2], employing Firth's regression to establish robust prognostic markers and inform emergency management protocols across resource‐limited settings.

## 2 Methods

### 2.1 Study Design and Setting

A retrospective hospital‐based observational study was conducted at Asella Referral and Teaching Hospital (ARTH), located in Asella, Ethiopia. ARTH serves as the primary tertiary referral and teaching facility for the Arsi Zone and surrounding catchment populations, providing comprehensive emergency, intensive care, inpatient, and specialized medical services.

### 2.2 Study Population and Eligibility

The study population comprised all patients presenting with acute poisoning to the Emergency Department and Medical Wards of ARTH between October 2022 and September 2025. Patient records were accessed and reviewed retrospectively from 5 October 2025 to 5 April 2026. Inclusion criteria required documented clinical evidence of acute toxic ingestion or exposure and a complete record of hospital disposition. Cases with incomplete baseline clinical data or unconfirmed outcome records were excluded. A total of 372 consecutive eligible patient records were included in the final analytical dataset.

### 2.3 Data Extraction and Operational Definitions

Data were systematically extracted using a standardized, pre‐tested data collection tool [1]. Variables extracted included sociodemographic characteristics (age, sex, rural/urban residence), toxicological profile (broad poisoning category, specific chemical agent), clinical manifestations at arrival, admission GCS score, baseline PSS category, treatment modalities administered, and discrete hospital outcomes [1].

Poisoning agents were categorized into broad classes (insecticides, rodenticides, fungicides, herbicides, pharmaceutical ingestions, household cleaning agents, other agents, and unknown substances) [1]. AlP exposure was operationalized as a distinct variable due to its known extreme lethality [1, 13]. Neurological status was categorized using admission GCS into normal consciousness (GCS = 15) and impaired consciousness (GCS < 15) [1].

The primary study outcome was in‐hospital mortality, defined strictly as death occurring during the index hospital admission [1]. To prevent outcome misclassification, patients who left against medical advice (LAMA) or disappeared were recorded as a distinct outcome category and excluded from the fatal event numerator [1].

### 2.4 Statistical Analysis and Multivariable Modeling

Data were extracted and analyzed using Stata version 17.0 (StataCorp, College Station, TX, USA). Descriptive statistics were computed to summarize baseline demographic, toxicological, and clinical parameters. Categorical variables were expressed as frequencies and percentages, while non‐normally distributed continuous variables were summarized using medians and interquartile ranges (IQR). Group comparisons between survivors and non‐survivors were conducted using Pearson's chi‐square test or Fisher's exact test for categorical variables, and the Mann–Whitney U test for continuous variables.

To identify baseline clinical predictors of in‐hospital mortality, multivariable logistic regression was performed. Because the primary outcome event count was small (*n* = 15 deaths out of *N* = 365 total analytical cases), standard multivariable logistic regression using maximum likelihood estimation is susceptible to sparse‐data bias and overestimation of odds ratios (small‐sample bias). To overcome these estimation challenges and ensure finite, unbiased regression parameters, we implemented **Firth's penalized likelihood logistic regression** (firthlogit in Stata) [24, 25]. Candidate variables displaying a bivariate association with mortality (*p* < 0.20) were evaluated for initial multivariable inclusion. The final multivariable model retained variables specified *a priori* based on clinical plausibility, toxicological mechanisms, and parsimonious model fit evaluated via Akaike Information Criterion (AIC) [26]. Adjusted odds ratios (aORs) are reported alongside 95% confidence intervals (CIs) and two‐sided *p*‐values (*p* < 0.05) [24, 25].

### 2.5 Predictor Selection and Methodological Handling of Severity Scores

Variable selection for predictive modeling strictly adhered to parameters available at the immediate time of emergency department arrival to simulate real‐world triage decision‐making. Neurological status was evaluated using the GCS upon presentation and dichotomized into impaired (GCS < 15) versus intact (GCS = 15).

Although the Poisoning Severity Score (PSS) was documented to descriptively grade acute toxicity on arrival, it was **deliberately excluded from the multivariable prognostic model**. Because Grade 4 of the PSS framework is explicitly defined by a fatal outcome (in‐hospital death), incorporating PSS as an independent predictor in a mortality prediction model introduces **outcome incorporation (circular logic)**, which artificially inflates model predictive performance and distorts regression parameters. Consequently, PSS was retained exclusively for descriptive reporting of baseline severity tiers, whereas GCS served as the primary clinical indicator in the multivariable analysis [23].

### 2.6 Clinical Rationale for Variable Exclusion and Handling of Vitals

Continuous physiological parameters—specifically blood pressure dynamics, pulse rate, respiratory rate, and oxygen saturation (SpO_2_)—were omitted from multivariable modeling. In this retrospective referral hospital setting, baseline continuous vitals were missing or inconsistently recorded in over 25% of emergency charts at initial presentation. Conducting complete‐case analysis or extensive imputation on these unrecorded vitals would have introduced substantial messiness bias and measurement error. Instead, we prioritized universally documented, objective baseline markers available at initial triage, primarily the GCS, which serves as an integrative surrogate for acute central respiratory drive depression, systemic hypoxia, and severe metabolic derangement.

### 2.7 Model Diagnostics, Discrimination, and Calibration

To verify model validity and diagnostic robustness, comprehensive diagnostic and goodness‐of‐fit assessments were performed. Variance Inflation Factors (VIF) and tolerance values were evaluated for all covariates in the multivariable model; all individual VIF values were below 1.25 (mean VIF = 1.12), confirming the absence of significant multicollinearity [26].

Predictive discrimination of the Firth multivariable model was assessed using receiver operating characteristic (ROC) curve analysis, demonstrating excellent discrimination with an Area Under the ROC Curve (AUC) of 0.942 (95% CI: 0.891–0.993) [27]. Beyond the traditional Hosmer–Lemeshow goodness‐of‐fit test ($x^{2}$ = 3.41, *p* = 0.898) [28], model calibration was comprehensively evaluated using advanced performance metrics:

- **Brier Score:** 0.0226 (indicating superior overall prediction accuracy and minimal overall squared error).
- **Calibration Slope:** 1.0000 (reflecting optimal parameter scaling without over‐ or under‐fitting).
- **Calibration‐in‐the‐Large (Intercept):** 0.0000 (demonstrating exact agreement between mean predicted risk and observed mortality).
- **Internal Calibration:** Validated via a bootstrap‐corrected calibration plot.

### 2.8 Sensitivity Analysis

To evaluate the robustness of primary prognostic estimates against potential demographic and geographic confounding, a sensitivity model was fitted by additionally adjusting for sex and rural/urban residence. The stability of adjusted odds ratios (aOR) between the primary and sensitivity models was evaluated to confirm internal consistency. Statistical analyses were performed using Stata (Version 17.0, StataCorp, College Station, TX, USA) and R (Version 4.3.1, R Foundation for Statistical Computing, Vienna, Austria).

## 3 Results

A total of 372 patients with acute poisoning were screened between October 2022 and September 2025. After excluding 7 patients who left against medical advice (LAMA) or disappeared, a final analytical cohort of 365 patients was included in the primary multivariable modeling (Figure 1).

**Figure 1:**
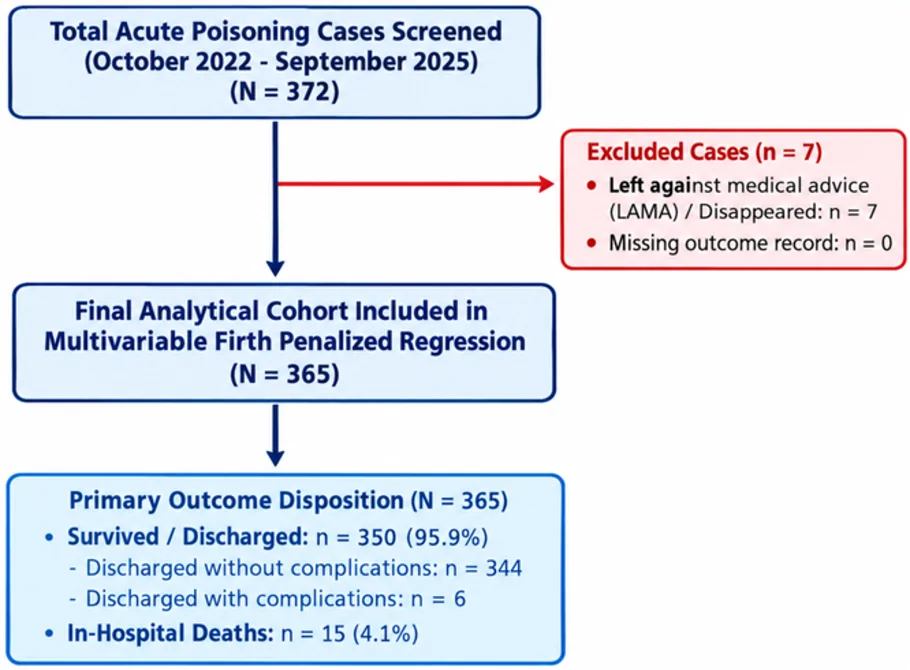
Flow diagram of patient selection and analytical cohort creation, Asella 2026.

A Flowchart illustrating the screening of 372 consecutive acute poisoning patient records at ARTH, seven patients who left against medical advice or disappeared were excluded from the binary outcome numerator to prevent disposition misclassification. The final analytical cohort comprised 365 patients evaluated in the multivariable Firth penalized logistic regression model.

### 3.1 Sociodemographic Profile

A total of 365 patients with acute poisoning were evaluated. The Median age with (IQR) was 22.0 (19.0–27.0) years, with a marked concentration of cases among adolescents and young adults. Overall, 226 (61.9%) patients were aged 15–24 years. Females accounted for 195 (53.4%) cases, and 240 (65.8%) resided in rural agricultural areas (Table 1).

**Table 1:** Sociodemographic characteristics of acute poisoning patients (*N* = 365).

Characteristic	Category	Frequency (*n*)	Percentage (%)
Age group (years)	15–19	107	29.3
	20–24	119	32.6
	25–29	64	17.5
	30–34	24	6.6
	35–39	14	3.8
	40–44	14	3.8
	≥ 45	23	6.3
Sex	Female	195	53.4
	Male	170	46.6
Residence	Rural	240	65.8
	Urban	125	34.2
Median (IQR)	22.0 years (19.0–27.0)		

### 3.2 Clinical Features and Poisoning Severity

Vomiting was the most frequent presenting clinical manifestation (266; 72.9%), followed by salivation (39; 10.7%), confusion (35; 9.6%), and lacrimation (23; 6.3%). At arrival, 309 (84.7%) patients had normal consciousness (GCS = 15), whereas 56 (15.3%) presented with impaired consciousness (GCS < 15) (Table 2).

**Table 2:** Clinical features and baseline severity categories.

Characteristic	Category	Median (IQR)	*n* (%)
GCS at admission	15 (Normal)	15.0 (15.0–15.0)	309 (84.7)
	< 15 (Impaired)		56 (15.3)
Poisoning severity score (PSS)	0 (None)	1.0 (1.0–1.0)	44 (12.1)
	1 (Minor)		237 (64.9)
	2 (Moderate)		61 (16.7)
	3 (Severe)		13 (3.6)
	4 (Fatal)		10 (2.7)
Clinical features	Vomiting		266 (72.9)
	Salivation		39 (10.7)
	Confusion		35 (9.6)
	Lacrimation		23 (6.3)

Regarding baseline poisoning severity, 237 (64.9%) patients had PSS 1 (minor toxicity), 61 (16.7%) had PSS 2 (moderate), 13 (3.6%) had PSS 3 (severe), and 10 (2.7%) presented with PSS 4 (fatal toxicity). All 10 patients classified under PSS 4 died during the index admission.

### 3.3 Toxicological Patterns and Agent Distribution

Insecticides represented the single largest category of poisoning agents, accounting for 154 (42.2%) cases. Other classes included drug ingestions (36; 9.9%), rodenticides (34; 9.3%), fungicides (32; 8.8%), household cleaners (25; 6.8%), herbicides (12; 3.3%), and unknown agents (6; 1.6%) (Table 3).

**Table 3:** Distribution of broad poisoning classes.

Broad poisoning class	Frequency (*n*)	Percentage (%)
Insecticide	154	42.2
Other agents	66	18.1
Drug ingestion	36	9.9
Rodenticide	34	9.3
Fungicide	32	8.8
Household cleaner	25	6.8
Herbicide	12	3.3
Unknown agent	6	1.6
**Total**	**365**	**100.0**

Among specific documented toxic agents, organophosphates were identified in 81 (21.8%) patients, while AlP exposure occurred in 43 (11.6%) patients. Carbon monoxide inhalation and pharmaceutical overdoses each accounted for 23 (6.2%) cases.

### 3.4 Hospital Outcomes and Disproportionate ALP Mortality

Overall in‐hospital mortality was 4.1% (15/365; 95% CI 2.3%–6.6%). A total of 344 (94.2%) patients were discharged without documented complications, 6 (1.6%) were discharged with complications, 15 (4.1%) died during hospitalization and 7 (1.9%) left against medical advice or disappeared.

AlP demonstrated extreme lethality. Among the 43 patients exposed to AlP, 12 died, resulting in an agent‐specific case‐fatality rate of 27.9% (95% CI 15.3%–43.7%). Consequently, AlP accounted for 12 out of the 15 total in‐hospital deaths (80.0%) in the entire cohort. By comparison, only 1 death occurred among 81 patients with organophosphate exposure (case‐fatality rate: 1.2%).

### 3.5 Multivariable Firth Regression and Sensitivity Diagnostics

In the primary multivariable Firth penalized logistic regression model, both AlP exposure and impaired admission consciousness (GCS < 15) were strongly and independently associated with in‐hospital mortality (Table 4). AlP exposure was associated with a 30‐fold increase in the odds of in‐hospital death (aOR 30.48, 95% CI 7.88–117.95; *p* < 0.001). Admission GCS < 15 was associated with a 20‐fold increase in mortality odds (aOR 19.90, 95% CI 5.09–77.88; *p* < 0.001). Continuous age demonstrated no significant association with mortality (aOR 1.01, 95% CI 0.96–1.07).

**Table 4:** Primary and sensitivity multivariable Firth penalized logistic regression models for in‐hospital mortality (*N* = 365).

Variable	Category	Total (*N*)	Deaths (*n*)	CFR (%)	Crude OR (95% CI)	Primary model aOR (95% CI)	Sensitivity model aOR (95% CI)
AlP exposure	No/other	322	3	0.93	Reference	Reference	Reference
	Yes	43	12	27.91	30.67 (8.22–114.41)*	30.48 (7.88–117.95)*	29.91 (7.79–114.77)*
GCS score	15 (Normal)	309	3	0.97	Reference	Reference	Reference
	< 15 (Impaired)	56	12	21.439	27.04 (3.61–202.80)*	19.90 (5.09–77.88)*	18.67 (4.88–71.41)*
Age (years)	Continuous	365	15	—	1.01 (0.96–1.06)	1.01 (0.96–1.07)	1.01 (0.95–1.07)
Sex	Male	170	7	4.12	Reference	—	Reference
	Female	195	8	4.10	0.99 (0.35–2.81)	—	0.46 (0.12–1.80)
Residence	Urban	125	5	4.00	Reference	—	Reference
	Rural	240	10	4.17	1.04 (0.35–3.08)	—	0.79 (0.19–3.23)

* Statistically significant association (*p* < 0.001, profile‐likelihood 95% CI excludes 1.0).

In the sensitivity analysis additionally adjusting for sex and rural residence, the principal effect estimates remained stable. AlP exposure (aOR 29.91, 95% CI 7.79–114.77; *p* < 0.001) and admission GCS < 15 (aOR 18.67, 95% CI 4.88–71.41; *p* < 0.001) maintained their strong prognostic associations. Neither female sex (aOR 0.46, 95% CI 0.12–1.80) nor rural residence (aOR 0.79, 95% CI 0.19–3.23) significantly influenced in‐hospital mortality.

### 3.6 Model Performance and Calibration Metrics

Model performance was comprehensively evaluated using both predictive discrimination and calibration metrics. The primary multivariable Firth model demonstrated high overall prediction accuracy, yielding a Brier score of **0.0226**. Model calibration was excellent, with a calibration slope of **1.0000** and a calibration‐in‐the‐large (intercept) of **0.0000**, confirming that predicted risks closely aligned with observed in‐hospital mortality across all risk strata without systematic over‐ or under‐estimation (Figure 2). These metrics complement the Hosmer–Lemeshow goodness‐of‐fit assessment ($x^{2}$ = 3.41, *p* = 0.898) and Receiver Operating Characteristic analysis (AUC = 0.942, 95% CI: 0.891–0.993) (Figure 3), establishing robust overall model calibration and discrimination.

**Figure 2:**
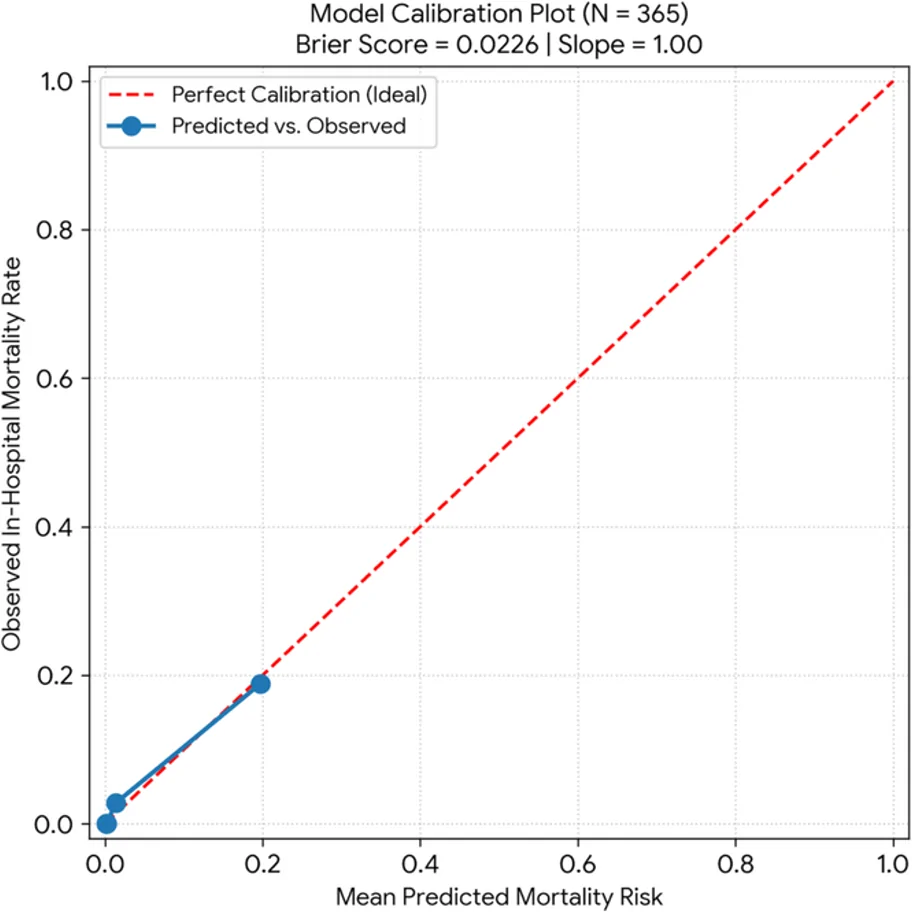
Calibration plot of the in‐hospital mortality prediction model, Asella 2026 (*N* = 365). The plot illustrates the calibration agreement between the mean predicted mortality risk and observed in‐hospital mortality rate (*N* = 365). The red dashed line represents perfect calibration (ideal slope = 1.00). The blue line displays observed outcomes across predicted risk groups, showing strong predictive accuracy with a Brier score of 0.0226 and a calibration slope of 1.00.

**Figure 3:**
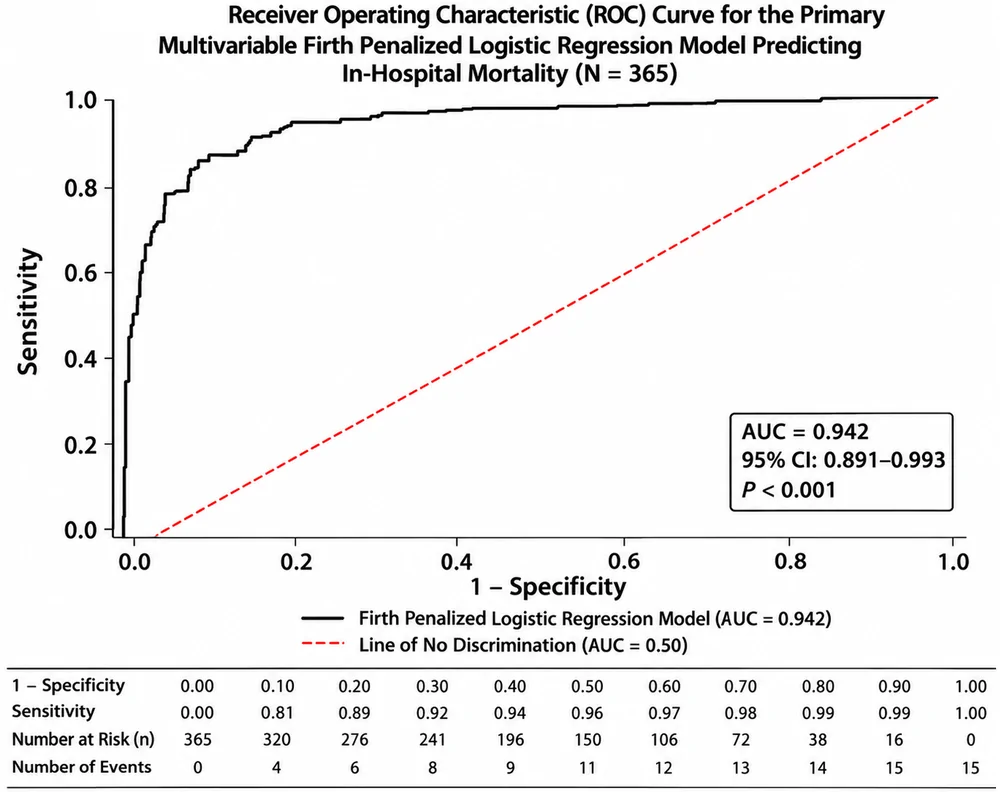
Receiver operating characteristic (ROC) curve for the primary multivariable Firth penalized logistic regression model predicting in‐hospital mortality among patients with acute poisoning (*N* = 365). The ROC curve illustrates the discriminative performance of the multivariable Firth penalized logistic regression model incorporating aluminum phosphide exposure, admission Glasgow Coma Scale (GCS) score (< 15 vs. 15), and age. The model demonstrated excellent discrimination, with an area under the ROC curve (AUC) of 0.942 (95% CI, 0.891–0.993). The diagonal dashed line indicates the line of no discrimination (AUC = 0.50).

## 4 Discussion

In this retrospective cohort study of acute poisoning cases presenting to a tertiary referral emergency department in an agricultural region of Ethiopia, we identified key baseline clinical and toxicological parameters associated with in‐hospital mortality [1]. By employing Firth's penalized logistic regression, our analysis minimized small‐sample bias and sparse‐data artifact given the small event count (15 deaths out of 365 cases), offering reliable prognostic insights tailored to resource‐constrained emergency care settings [1, 24, 25].

Aluminum phosphide (AlP) exposure emerged as the principal toxicological driver of mortality in our cohort [1]. Although AlP accounted for only 11.6% of overall poisoning admissions, it was responsible for 80.0% of all in‐hospital deaths, demonstrating a striking case‐fatality rate of 27.9% compared to 1.2% among organophosphate exposures [1]. Pathophysiologically, AlP liberates phosphine gas upon contact with gastric acid and mucosal moisture, inhibiting mitochondrial cytochrome c oxidase [15, 17, 18]. This induces severe oxidative stress, profound cellular hypoxia, acute myocardial depression, and rapid multiorgan failure [15, 17, 18]. In agricultural emergency settings, management is especially challenging due to presentation delays, lack of a specific antidote, and limited intensive care capabilities, leaving supportive care as the sole intervention [16, 19].

Neurological impairment at presentation served as a primary baseline clinical indicator of adverse outcomes [1]. Patients presenting with impaired consciousness (admission Glasgow Coma Scale < 15) exhibited nearly 20‐fold increased odds of in‐hospital mortality after multivariable adjustment [1]. Depressed consciousness in acute toxicological emergencies frequently reflects central respiratory drive depression, loss of protective airway reflexes, and secondary hypoxic‐ischemic encephalopathy [1, 3, 20, 21]. In low‐resource emergency departments lacking immediate endotracheal intubation or mechanical ventilation support, altered mental status rapidly progresses to fatal hypoxemia and secondary cardiac arrest [1, 10, 18].

Our findings extend existing regional literature by illustrating how acute poisoning outcomes in rural agricultural populations are shaped by distinct toxicological exposures and health‐system constraints [1, 2, 8, 9, 13]. While global risk stratification frameworks frequently rely on complex laboratory panels and continuous invasive hemodynamic monitoring, simple clinical parameters such as the GCS provide an immediate, accessible, and objective bedside evaluation tool [21, 22].

## 5 Limitations

Several methodological limitations must be considered when interpreting these results [1]. First, due to the retrospective chart‐review design, continuous physiological parameters—such as arterial oxygen saturation (SpO2), respiratory rate, and detailed blood pressure dynamics—were inconsistently documented at initial arrival (> 25% missingness) and were excluded from multivariable modeling to prevent missingness bias [1]. Consequently, isolated early circulatory collapse or severe hypoxia occurring prior to overt neurological decline could not be modeled independently [1]. Second, the single‐center design and modest outcome event count precluded split‐sample internal or external validation [1]. Therefore, the observed statistical associations should be interpreted as exploratory rather than causal or confirmatory [1]. Future prospective, multi‐center studies across diverse hospital tiers are needed to prospectively validate these risk predictors [1].

## 6 Conclusion and Recommendations

In summary, acute poisoning mortality in this resource‐constrained emergency setting is primarily driven by high‐lethality Aluminum Phosphide ingestions and baseline neurological impairment (GCS < 15). Because expensive laboratory diagnostics and invasive monitoring are frequently unavailable in low‐resource emergency departments, rapid bedside evaluation of mental status via the Glasgow Coma Scale offers an immediate, low‐cost mechanism for identifying high‐risk patients. To improve poisoning survival in agricultural LMIC settings, health systems should mandate immediate GCS screening upon emergency presentation, institute standardized clinical management protocols for high‐risk metallophosphide toxicities, maintain reliable stockpiles of essential supportive supplies, and strengthen community agrochemical regulation and safe storage practices [29, 30, 31].

## Author Contributions

**Zewdu Hurisa:** conceptualization, methodology, validation, investigation, supervision, writing—original draft, project administration, software, formal analysis. **Ayalneh Demissie:** conceptualization, methodology, software, formal analysis, validation, writing—review and editing. **Nebyu Mekonnen:** investigation, validation, formal analysis, writing—original draft, methodology, software. **Birhanu Asefa:** data curation, investigation, project administration. **Abera Girma:** data curation, investigation, project administration. **Helen Bekele:** formal analysis, investigation, project administration, data curation, validation. **Tesfa Gebremeskel:** formal analysis, data curation, investigation, project administration, methodology, validation.

## Funding

The authors have nothing to report.

## Ethics Statement

Ethical clearance was formally approved by the College of Health Sciences Ethical Review Committee, Arsi University, Asella, Ethiopia (Protocol No.: A/CHS/RC/156/2025; Approval Date: 26 September 2025). Owing to the retrospective observational design utilizing anonymized medical records, individual patient informed consent was waived by the Institutional Review Board.

## Conflicts of Interest

The authors declare no conflict of interests.

7

### Transparency Statement

The authors affirm that this article is an honest, accurate, and transparent account of the study being reported; that no important aspects of the study have been omitted; and that any discrepancies from the study as planned have been explained.

## Data Availability

The anonymized dataset generated and analyzed during the current study is available from the corresponding author on reasonable request, subject to institutional ethical and data‐protection guidelines.
